# *RNY4* in Circulating Exosomes of Patients With Pediatric Anaplastic Large Cell Lymphoma: An Active Player?

**DOI:** 10.3389/fonc.2020.00238

**Published:** 2020-02-27

**Authors:** Federica Lovisa, Piero Di Battista, Enrico Gaffo, Carlotta C. Damanti, Anna Garbin, Ilaria Gallingani, Elisa Carraro, Marta Pillon, Alessandra Biffi, Stefania Bortoluzzi, Lara Mussolin

**Affiliations:** ^1^Clinic of Pediatric Onco-Hematology, Department of Women's and Children's Health, University of Padova, Padova, Italy; ^2^Istituto di Ricerca Pediatrica Città della Speranza, Padova, Italy; ^3^Department of Molecular Medicine, University of Padova, Padova, Italy; ^4^Gene Therapy Program, Dana Farber/Boston Children's Cancer and Blood Disorders Centers, Boston, MA, United States; ^5^CRIBI Interdepartmental Research Center for Innovative Biotechnologies (CRIBI), University of Padova, Padova, Italy

**Keywords:** ALCL, liquidbiopsy, exosomes, YRNA, RNA-seq, small RNA

## Abstract

Emerging evidence indicates that extracellular vesicles, particularly exosomes, play a role in several biological processes and actively contribute to cancer development and progression, by carrying and delivering proteins, transcripts and small RNAs (sRNAs). There is high interest in studying exosomes of cancer patients both to develop non-invasive liquid biopsy tests for risk stratification and to elucidate their possible involvement in disease mechanisms. We profiled by RNA-seq the sRNA content of circulating exosomes of 20 pediatric patients with Anaplastic Large Cell Lymphoma (ALCL) and five healthy controls. Our analysis disclosed that non-miRNA derived sRNAs constitute the prominent fraction of sRNA loaded in exosomes and identified 180 sRNAs significantly more abundant in exosomes of ALCL patients compared to controls. YRNA fragments, accounting for most of exosomal content and being significantly increased in ALCL patients, were prioritized for further investigation by qRT-PCR. Quantification of *RNY4* fragments and full-length sequences disclosed that the latter are massively loaded into exosomes of ALCL patients with more advanced and aggressive disease. These results are discussed in light of recent findings on the role of *RNY4* in the modulation of tumor microenvironment.

## Introduction

Extracellular vesicles (EVs) are cell-derived membrane particles secreted from many cell types and circulating in body fluids, including plasma. Among different classes of EVs of different size and intracellular origin, exosomes are 40–150 nm endosome-derived EV originating from the inward budding of the limiting membrane of multivesicular bodies (1). In this process, exosomes are packed with proteins, lipids, DNAs, messenger RNAs (mRNAs) and non-coding RNAs, which can be transferred to recipient cells, and function both as paracrine and endocrine factors (2).

A large body of evidence collected in the last years proved the functional involvement of exosomes in cancer progression and spreading, induction of angiogenesis, as well as in chemoresistance and immune response evasion during tumor development (3). In this scenario, defining the peculiarities of exosomal cargo in cancer patients is a hot topic in biomedical research. The characterization of small non-coding RNAs (sRNAs) in plasmatic exosomes of cancer patients attracted interest for the identification of non-invasive disease biomarkers and, notably, in consideration of sRNA regulatory functions and their direct involvement in cancer mechanisms (4, 5).

Most functional studies on circulating sRNAs carried by tumor-derived exosomes were focused on microRNAs (miRNAs) because of their well-characterized regulatory roles in key signaling axes: exosome-delivered miRNAs have been shown to promote epithelial-mesenchymal transition (6, 7), induce angiogenesis and increase vascular leakage (8–10), prepare pre-metastatic niches to promote metastasis (11, 12) or induce tumor resistance to immune responses (13, 14).

Non-Hodgkin lymphoma (NHL) is a heterogeneous group of lymphoid malignancies and the fourth most common malignancy across the pediatric age spectrum. Considerable progress has been achieved in developing curative therapy for pediatric NHL, with an overall survival rate now exceeding 80% (15). There are three major categories of NHL: mature B-cell neoplasms, Lymphoblastic Lymphoma, and Anaplastic Large Cell Lymphoma (ALCL). Other NHL subtypes, including peripheral T-cell lymphomas, follicular lymphomas, and rare entities, represent <3% of the cases (16).

ALCL accounts for 10–15% of pediatric and adolescent NHL. Differently from ALCL in adults, ALCL in children is nearly universally ALK-positive and, in almost all of the cases, it is characterized by the *t*(2;5)(p23;q35) translocation, which leads to the constitutive expression of the NPM-ALK fusion protein (17). Although current treatment strategies achieve an event-free survival (EFS) of ~75% after 5 years, about 30% of the patients are resistant to therapy or experience a relapse (18). In this clinical context, new disease biomarkers are needed to enable the early identification of high-risk patients and a better tailoring of treatment. In ALCL, the identification of new active players in lymphomagenesis and in cancer cells dissemination mechanisms would have high potential for the design of innovative therapeutic interventions.

Currently, only a few, mainly descriptive, studies reported data regarding the sRNA cargo of lymphoma-derived exosomes and EVs. Moreover, only Diffuse Large B-cell Lymphoma (DLBCL), which is the most frequent histological subtype presenting in adults (19, 20), was investigated.

Studies aiming at the identification of clinically relevant sRNAs in plasmatic exosomes/EVs from lymphoma patients focused on miRNAs (21). The first evidence on EV miRNAs as a molecular diagnostic tool for disease monitoring in Hodgkin lymphoma (HL) patients was reported by Eijndhoven et al. (22). Specifically, lymphoma-associated miR-21-5p, miR-127-3p, miR-24-3p, let-7a-5p, and miR-155-5p were significantly increased in plasmatic EV from HL patients compared to healthy donors (HD). Exosome-derived miRNAs were also proposed as predictive biomarkers of chemotherapy resistance in DLBCL, where increased levels of miR-99a-5p and miR-125b-5p in patient plasmatic exosomes were associated with reduced progression-free survival (23). Data on exosomal sRNAs in pediatric lymphomas setting are currently missing.

Further, our appreciation of the small transcriptome complexity largely increased in the last years. Alternative processing of miRNA precursors (24–26) and housekeeping non-coding RNAs can generate miRNA-like sRNAs that can be functional and play roles in malignancies (27, 28). It's worth noting that several RNA-seq studies on exosomes derived from both tumoral and non-tumoral cell lines revealed that, differently from secreting cells, miRNAs constitute a minor percentage of EV-enclosed RNA. Besides protein coding mRNA, the EV fractions contain vault RNAs, YRNAs, small nuclear and nucleolar RNAs (snRNAs and snoRNAs), transfer RNAs (tRNA), as well as fragments deriving from long non-coding RNAs and transcribed pseudogenes (29–31).

In this perspective article, we present original data about the characterization of exosomal sRNAs in pediatric ALCL aiming attention on non-miRNA derived sRNAs, and discuss them in the frame of current literature, providing an original viewpoint of the possible translational relevance of these findings.

## *RNY4* Fragment Enrichment In Circulating Exosomes Of Alcl Patients Discovered By RNA-seq

To characterize the exosomal load of sRNAs in ALCL patients and disclose differences with healthy donors, sRNAs were examined by small RNA-seq of exosomes from 20 ALK-positive ALCL patients and five HD plasma samples, and from supernatant of five ALCL cell lines (Karpas299, SUDHL1, and SUP-M2, ALK-positive; FE-PD and MAC2A, ALK-negative). For patients, paired biopsy samples, all positive for the NPM-ALK fusion, were also sequenced. Exosomal RNA was extracted by using exoRNeasy Maxi/Midi kit (Qiagen) and assessed for proper amount and quality by Agilent 2100 Bioanalyzer (Agilent Technologies). RNA-seq libraries were prepared with NEBNext Multiplex Small RNA Library Prep Kit for Illumina (New England Biolabs), as previously reported (32), and sequenced on an Illumina HiSeq 4000 platform with single-end reads and average depth of 15 and 30 M for exosomal and biopsy samples, respectively.

After a preprocessing phase for adapter trimming and selection of high-quality reads (Qphred ≥ 30), data underwent analysis by miR&moRe software (24, 25). Briefly, miR&moRe maps filtered reads to genome assembly and the known hairpins sequences from miRBase extended in either directions by additional 30 bp and allows detection and prediction of miRNA hairpins and of the corresponding mature forms, as in Gaffo et al. (33). MiR&moRe allowed identification and quantification of 1,194 and 523 miRNA-derived sRNA species in biopsies and exosomal samples, including miRNAs and microRNA-offset RNAs derived from annotated and newly predicted miRNA precursors.

**Figure 1:**
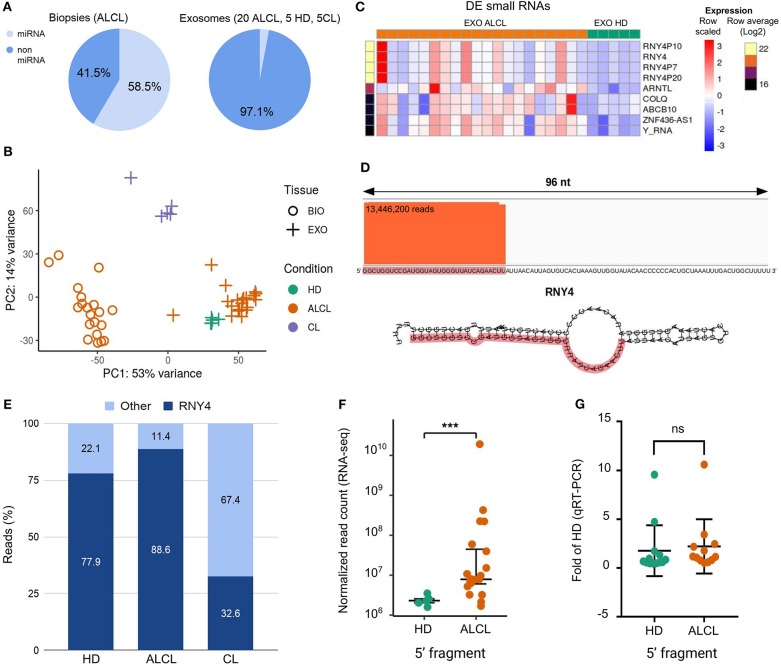
Characterization of exosomal small RNAs in ALCL patients. **(A)** Average proportion of sequence reads aligned to and outside miRNA precursors in anaplastic large cell lymphoma (ALCL) primary tumor biopsies, and in exosomes from ALCL patients, healthy donors (HD) and ALCL cell lines (CL). **(B)** Principal component analysis computed on expression profiles of the 1,007 most abundant (top 5% of expression in at least one sample) non miRNA-derived sRNAs. **(C)** Heatmap of expression profiles in exosome samples of the nine sRNAs most abundant (top 1%) and most varied comparing ALCL and HD (LFC>2). **(D)** Sequence reads alignment to *RNY4* shows reads stacked on the *RNY4* 5′ end; *RNY4* predicted secondary structure is displayed below, highlighting in red the 32 nt fragment identified through RNA-seq. **(E)** Average percentages of non-miRNA RNA-seq reads from exosomes samples mapping to *RNY4* locus and to other putative sRNAs. **(F)** Box-plot of *RNY4*-5′F expression in HD (*n* = 5) and ALCL (*n* = 20) exosomes, according to RNA-seq data. **(G)** Box-plot of *RNY4*-5′F expression measured by qRT-PCR in HD (*n* = 13) and ALCL (*n* = 12) exosomes [***DESeq2 p.adj < 0.001 independent samples from those shown in **(F)**, expression in ALCL relative to average in HD; comparative delta Ct method (2^−ΔΔCt^); miR-26a-5p used as endogenous control; Mann-Whitney].

We observed that in biopsies ~59% of the reads derived from miRNAs, whereas the large majority of the reads from exosomal samples did not align to known nor predicted miRNA precursors, concordantly in exosomes isolated from ALCL patients (2.5%), cell lines (2.6%), and healthy donors (5.3%) (Figure 1A). A similarly large fraction of non-miRNA sRNAs in EVs was described by other studies on lymphoma, melanoma, breast cancer and immune cells (19, 20, 29–31).

The non-miRNA sRNAs fraction in exosomes of ALCL patients was further characterized. First, reads not aligned to known or predicted miRNA precursors were mapped with Bowtie v1.1.2 (34) to the reference genome, allowing no mismatches and up to 15 multiple alignments. The alignments were then analyzed with *derfinder* software tool (35) to identify expressed RNAs and the corresponding genomic regions. Among the 9,181 regions supported by at least 10 reads we selected those of 13–50 contiguous bases, consistently with the size of sequencing library fragments. After expression normalization [DESeq2 (36)], 1,007 most abundant (top 5% of expression) putative non-miRNA-derived sRNAs were considered for further characterization. Principal component analysis of expression profiles of these 1,007 sRNAs clearly distinguished tissue from exosome samples, separating as well as exosomes from healthy donors, patients and cell lines (Figure 1B).

Next, 180 sRNAs were identified with significantly different abundance between exosomes of ALCL patients and HD (DESeq2 p.adj < 0.001). The heatmap in Figure 1C shows the expression of the nine sRNAs with highest abundance and increase in ALCL exosomes. Apparently, the sRNA present in greater supply in ALCL exosomes was defined by reads aligned to the *RNY4* gene, and to highly similar pseudogenes (*RNY4P7, RNY4P10*, and *RNY4P20*). Precisely, a fragment corresponding to the first 32 bases on the 5′ end of *RNY4* (*RNY4*-5′F) (Figure 1D) accounted for at least 80% of the non-miRNA sRNA expression in exosomes, whereas it was less abundant in exosomes derived from the cell line supernatant, isolated with the same protocol (Figure 1E). Remarkably, *RNY4*-5′F was five times and significantly more abundant in ALCL than in HD exosomes (p.adj = 0.0003; Figure 1F). The expression of *RNY4*-5′F was assessed by quantitative real-time PCR (qRT-PCR; Custom TaqMan Small RNA assay designed on the 32 bases of the *RNY4* sequence; ThermoFisher Scientific, Life Technologies) in 25 independent samples (12 ALCL and 13 HD). No difference was observed comparing exosomes from ALCL and HD (Figure 1G) not confirming RNA-seq result (Figure 1F).

YRNAs were first discovered in 1981 as 83–112 nt RNA components of circulating ribonucleoproteins, complexed to Ro60 and La autoantigens, in serum of patients with autoimmune diseases (37, 38). Evolutionary conserved in vertebrates (39), YRNAs fold in characteristic stem-loop secondary structures, with lower and upper stem loop sequences being the most conserved (40). Four different human YRNAs (*RNY1, RNY3, RNY4*, and *RNY5*) are transcribed in the nucleus by RNA polymerase III from genes clustered together at a single locus on chromosome 7q36 (41). Intracellularly, binding of the lower YRNA stem to Ro60 was shown to be involved in the maintenance of RNA stability and in cellular response to stress (42), whereas the upper stem was proven to be essential for the initiation of chromosomal DNA replication (43). In addition, YRNAs were also linked to alternative splicing and regulation of the translation of specific RNAs, since most YRNA-associated proteins are implicated in these processes (44).

In recent years, YRNAs and YRNA fragments derived from site-specific cleavage by RNase L were reported to be enriched in different types of EV compared to secreting cancer cells (19, 31, 45–47). Noteworthy, RNA fragments corresponding to the 5′ region of the *RNY4*, almost exactly corresponding to the *RNY4*-5′F detected in the present study, were shown to be the most abundant sRNA species in plasma samples from HD (30, 48–50) and melanoma patients (51), as well as in breast cancer patients' exosomes and plasma (30, 49), and plasmatic exosomes from non-small cell lung cancer (NSCLC) and chronic lymphocytic leukemia (CLL) patients (52, 53).

Since YRNA fragments derive from conserved ends of the YRNA hairpin, it was initially hypothesized that YRNAs could “conceal miRNAs” and be processed in miRNA-sized YRNA fragments that could function as miRNAs (54). However, this hypothesis was not supported by later studies. Since YRNA fragment biogenesis resulted to be Dicer-independent, they were found in complexes different from those associated with microRNAs and they did not co-immunoprecipitate with Ago2 (55). Moreover, they did not regulate targets tested by Thomson and colleagues in a miRNA-like manner (56).

Recently, since *RNY5* fragments administration to human primary fibroblasts was shown to induce cell death (46) a role for *RNY3* in enhancing “cleavage and polyadenylation specificity factor” (CPSF) recruitment to histone locus bodies has been proposed (57), thus associating YRNA fragments to functional activities different from those typical of miRNAs.

## Genuine miRNA-Like *RNY4* Fragments OR Full-Length *RNY4*?

The increased levels of *RNY4*-derived fragments or full-length transcripts circulating in plasma or exosomes of cancer patients compared to HD (30, 49, 53) triggered interest in the potential use of *RNY4* as a cancer biomarker. However, whether *RNY4*-derived fragments detected by small RNA-seq are genuine fragments or reflect the presence of the full-length *RNY4* is still a matter of debate.

By Northern blotting, Dhahbi et al. confirmed 5′ *RNY4* fragments in plasma of HD (48), Haderk et al. validated the presence of both *RNY4* full-length and 5′ fragments in CLL exosomes (53), whereas Driedonks et al. showed that EV released from dendritic cells mostly contain full-length *RNY1* and only small amounts of 19–35 nt *RNY1* fragments (58). In this regard, Driedonks and Nolte-'t Hoen suggested that YRNA secondary structures might impede full-length cDNA synthesis, leading to overestimation of fragmented non-coding RNA in sequencing data (59). Indeed, in a RNA-seq study, Godoy et al. detected fragments derived from both the 5′ and 3′ arms (60) whereas other works reported mostly full-length YRNAs (61, 62).

**Figure 2:**
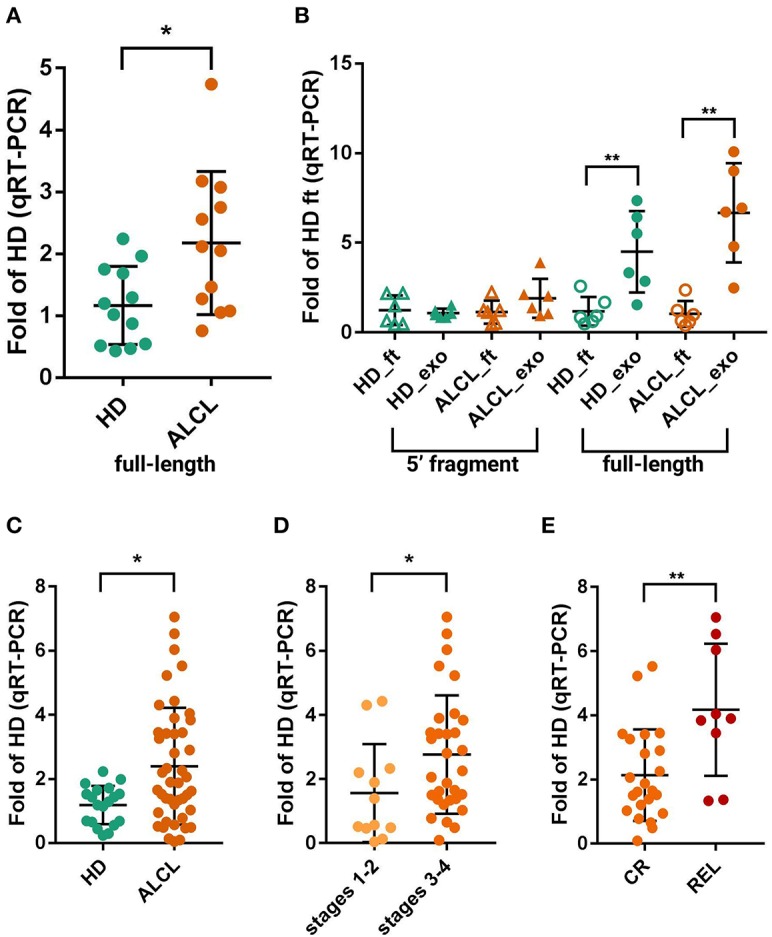
Quantification of *RNY4*-5′F and full-length *RNY4* by qRT-PCR. **(A)** Expression of full-length *RNY4* in 12 HD and 12 ALCL plasmatic exosomes measures by qRT-PCR. **(B)** Expression of *RNY4*-5′F (5′ fragment) and full-length *RNY4* loaded into exosomes (exo) and freely circulating in plasma (flow-through, ft); expression in ALCL relative to average in HD_ft. Full-length *RNY4* was significantly more abundant in both HD and ALCL exosomes than as free circulating RNA (6 HD, 6 ALCL), whereas the *RNY4*-5′F was almost equally distributed inside exosomes and as free circulating sRNA in both HD and ALCL. **(C)** Evaluation of full-length *RNY4* in exosomes from an extended cohort of 44 ALCL and 19 HD confirmed a significantly increased expression of the full-length form in ALCL compared to HD. Full-length *RNY4* was significantly more abundant in patients with stage 3–4 disease compared to those in stages 1–2 **(D)** and also among stage 3–4, in relapsed patients (REL) compared to those in stable complete remission (CR) **(E)**. For panels **(A,C,D,E)**, expression in ALCL has been calculated relative to average in HD; comparative delta Ct method (2^−ΔΔCt^), miR-26a-5p as endogenous control, Mann-Whitney test for sample comparison were used for all panels. (*0.01 < *p* < 0.05, **p < 0.01).

To verify if full-length *RNY4* might be responsible for the differential expression detected by small RNA-seq, not validated by RT-PCR specific from *RNY4*-5′F, we quantified the full-length *RNY4* in 12 ALCL and 12 HD plasmatic exosomes by qRT-PCR using primers from Tolkach et al. (63). The full-length *RNY4* was significantly more abundant in ALCL than in HD (Mann Whitney, *p* = 0.017) (Figure 2A).

Further, we considered that full-length YRNAs were mainly reported as contained in exosomes (59), whereas fragments were previously validated by Northern blotting in total plasma and in exosomes (48, 53). We thus investigated the amount of full-length *RNY4* and the *RNY4*-5′F, also distinguishing RNA loaded into exosomes and freely circulating in plasma (RNA of six HD and six ALCL from both exosomes and the column flow-through after the exosome binding step). Of note, most of the full-length *RNY4* was inside exosomes (5.6 times more abundant inside, on average) of both HD (Mann-Whitney *p* = 0.009) and ALCL patient (*p* = 0.002) samples, whereas the fragment was present at a similar level in exosomes and as free circulating RNA (Figure 2B). Taken together, these results suggested that *bona fide RNY4* fragments circulate in plasma both enclosed in membranes and as free RNAs, in amounts not discriminating ALCL and HD, whereas full-length *RNY4* is mainly enclosed in exosomes, where it is significantly enriched in ALCL patients.

A further examination of an extended cohort of 44 ALCL and 19 HD plasmatic exosomes confirmed the upregulation of the full-length form in ALCL samples (Mann Whitney, *p* = 0.017) (Figure 2C).

## Full-Length *RNY4* Load In Exosomes Of ALCL Patients Correlates with Disease Aggressiveness

Next, the extended cohort was further examined considering clinical data. Of importance, *RNY4* abundance correlated with ALCL patients' clinical characteristics. The full-length *RNY4* was more abundant in exosomes of ALCL patients with advanced disease stages (32 ALCL in 3–4 stage vs. 12 ALCL in 1–2 stage; Mann Whitney, *p* = 0.049) (Figure 2D). Since most (9/10) of the relapsed patients were diagnosed in stages 3–4 (Figure 2D), we analyzed *RNY4* amount at diagnosis in relation to relapse, considering only advanced stages. Compared to cases in stable complete remission (*N* = 23), relapsed patients (*N* = 9) presented at diagnosis with increased levels of exosomal full-length *RNY4* (Mann Whitney, *p* = 0.0065) (Figure 2E).

These findings indicate exosomal *RNY4* as a promising biomarker of disease aggressiveness in ALCL, to be quantified with a simple and non-invasive liquid biopsy. Moreover, our data and literature evidence collectively encourage further investigation to ascertain a possible functional role of *RNY4* in ALCL disease aggressiveness, as well as in other lymphoproliferative diseases or different malignancies. Indeed, *RNY4* delivery by CLL exosomes has been recently shown to induce key leukemia-associated phenotypes in monocytes, such as the release of pro-tumorigenic cytokines (CCL2, CCL4, and IL-6) and the expression of the immunosuppressive protein PD-L1, thus generating a tumor-supporting microenvironment (53). ALCL tumors are characterized by variable histological patterns, mostly depending on tumor cell size and the presence of a large number of reactive histiocytes in the background (64). The biological functions of YRNAs could be multidirectional and we speculate that the association between ALCL and changes in these circulating YRNA reflects some aspects of either the biology of the tumor or the immunosystem reaction of the individual to the tumor. In particular, our results pave the way for investigating the role of *RNY4* as mediator of immunoescape in lymphoma patients. The treatment of monocytes *ex vivo* with tumor exosomes, the uptake as well as exosome-mediated responses by flow cytometry, or cytokine quantification can be used in the next future to elucidate this intriguing aspect.

In conclusion, *RNY4* is a massively loaded molecule in exosomes of ALCL patients, with *RNY4* significantly increased in patients compared to controls. Notably, significantly higher *RNY4* levels were observed in patients diagnosed at advanced stages, and among them, in those that later relapsed. These findings, in the light of available functional data on exosomal *RNY4*, encourage further study of *RNY4* involvement in ALCL tumor microenvironment and disease aggressiveness.

## Data Availability Statement

Publicly available datasets were analyzed in this study, these can be found in the NCBI Gene Expression Omnibus (GSE144781).

## Ethics Statement

The study was approved by the ethics committee for clinical experimentation of the Padova hospital CESC (Comitato Etico per la sperimentazione clinica azienda ospedaliera di Padova). Written informed consent was obtained from patients and/or their legal guardians in accordance with the institution's ethical review boards.

## Author Contributions

FL designed the RNA-seq experiment and the experimental work, analyzed data, and wrote the manuscript. PD performed RNA-seq data analysis and qRT-PCR, and contributed to wrote the manuscript. EG contributed to RNA-seq data analysis and revised the manuscript. PD, FL, and EG prepared figures. CD, AG, and IG processed clinical samples and performed qRT-PCR. EC and MP collected clinical data and commented on manuscript. AB revised the manuscript. SB conceived the study, supervised the bioinformatics work, contributed to experimental results interpretation, and wrote the manuscript. LM conceived the study, supervised the experimental work, and revised the manuscript.

### Conflict of Interest

The authors declare that the research was conducted in the absence of any commercial or financial relationships that could be construed as a potential conflict of interest.

## References

[1] Abels ER Breakefield XO. Introduction to extracellular vesicles: biogenesis, RNA cargo selection, content, release, and uptake. Cell Mol Neurobiol. (2016) 36:301–12. 10.1007/s10571-016-0366-z 27053351 PMC5546313

[2] Maia J Caja S StranoMoraes MC Couto N Costa-Silva B. Exosome-based cell-cell communication in the tumor microenvironment. Front Cell Dev Biol. (2018) 6:18. 10.3389/fcell.2018.00018 29515996 PMC5826063

[3] Mashouri L Yousefi H Aref AR Ahadi AM Molaei F Alahari SK. Exosomes: composition, biogenesis, and mechanisms in cancer metastasis and drug resistance. Mol Cancer. (2019) 18:75. 10.1186/s12943-019-0991-5 30940145 PMC6444571

[4] Esteller M. Non-coding RNAs in human disease. Nat Rev Genet. (2011) 12:861–74. 10.1038/nrg3074 22094949

[5] Anastasiadou E Jacob LS Slack FJ. Non-coding RNA networks in cancer. Nat Rev Cancer. (2018) 18:5–18. 10.1038/nrc.2017.99 29170536 PMC6337726

[6] Xiao D Barry S Kmetz D Egger M Pan J Rai SN. Melanoma cell-derived exosomes promote epithelial-mesenchymal transition in primary melanocytes through paracrine/autocrine signaling in the tumor microenvironment. Cancer Lett. (2016) 376:318–27. 10.1016/j.canlet.2016.03.050 27063098 PMC4869527

[7] Bigagli E Luceri C Guasti D Cinci L. Exosomes secreted from human colon cancer cells influence the adhesion of neighboring metastatic cells: role of microRNA-210. Cancer BiolTher. (2016) 17:1062–9. 10.1080/15384047.2016.1219815 27611932 PMC5079399

[8] Bao L You B Shi S Shan Y Zhang Q Yue H. Metastasis-associated miR-23a from nasopharyngeal carcinoma-derived exosomes mediates angiogenesis by repressing a novel target gene TSGA10. Oncogene. (2018) 37:2873–89. 10.1038/s41388-018-0183-6 29520105 PMC5966363

[9] Fang J-H Zhang Z-J Shang L-R Luo Y-W Lin Y-F Yuan Y. Hepatoma cell-secreted exosomal microRNA-103 increases vascular permeability and promotes metastasis by targeting junction proteins. Hepatology. (2018) 68:1459–75. 10.1002/hep.29920 29637568

[10] Zeng Z Li Y Pan Y Lan X Song F Sun J. Cancer-derived exosomal miR-25-3p promotes pre-metastatic niche formation by inducing vascular permeability and angiogenesis. Nat Commun. (2018) 9:5395. 10.1038/s41467-018-07810-w 30568162 PMC6300604

[11] Ono M Kosaka N Tominaga N Yoshioka Y Takeshita F Takahashi R-U. Exosomes from bone marrow mesenchymal stem cells contain a microRNA that promotes dormancy in metastatic breast cancer cells. Sci Signal. (2014) 7:ra63. 10.1126/scisignal.2005231 24985346

[12] Fong MY Zhou W Liu L Alontaga AY Chandra M Ashby J. Breast-cancer-secreted miR-122 reprograms glucose metabolism in premetastatic niche to promote metastasis. Nat Cell Biol. (2015) 17:183–94. 10.1038/ncb3094 25621950 PMC4380143

[13] Ye S-B Li Z-L Luo D-H Huang B-J Chen Y-S Zhang X-S. Tumor-derived exosomes promote tumor progression and T-cell dysfunction through the regulation of enriched exosomal microRNAs in human nasopharyngeal carcinoma. Oncotarget. (2014) 5: 5439–52. 10.18632/oncotarget.2118 24978137 PMC4170615

[14] Hsu Y-L Hung J-Y Chang W-A Jian S-F Lin Y-S Pan Y-C. Hypoxic lung-cancer-derived extracellular vesicle microRNA-103a increases the oncogenic effects of macrophages by targeting PTEN. Mol Ther. (2018) 26:568–81. 10.1016/j.ymthe.2017.11.016 29292163 PMC5835028

[15] Minard-Colin V Brugières L Reiter A Cairo MS Gross TG Woessmann W. Non-Hodgkin lymphoma in children and adolescents: progress through effective collaboration, current knowledge, and challenges ahead. J Clin Oncol. (2015) 33:2963–74. 10.1200/JCO.2014.59.5827 26304908 PMC4979194

[16] Arber DA Orazi A Hasserjian R Thiele J Borowitz MJ Le Beau MM. The 2016 revision to the World Health Organization classification of myeloid neoplasms and acute leukemia. Blood. (2016) 127:2391–405. 10.1182/blood-2016-03-643544 27069254

[17] Turner SD Lamant L Kenner L Brugières L. Anaplastic large cell lymphoma in paediatric and young adult patients. Br J Haematol. (2016) 173:560–72. 10.1111/bjh.13958 26913827

[18] Le Deley M-C Reiter A Williams D Delsol G Oschlies I McCarthy K. Prognostic factors in childhood anaplastic large cell lymphoma: results of a large European intergroup study. Blood. (2008) 111:1560–6. 10.1182/blood-2007-07-100958 17957029

[19] Koppers-Lalic D Hackenberg M Bijnsdorp IV van Eijndhoven MAJ Sadek P Sie D. Nontemplated nucleotide additions distinguish the small RNA composition in cells from exosomes. Cell Rep. (2014) 8:1649–58. 10.1016/j.celrep.2014.08.027 25242326

[20] Rutherford SC Fachel AA Li S Sawh S Muley A Ishii J. Extracellular vesicles in DLBCL provide abundant clues to aberrant transcriptional programming and genomic alterations. Blood. (2018) 132:e13–23. 10.1182/blood-2017-12-821843 29967128 PMC6265635

[21] Li J Tian T Zhou X. The role of exosomal shuttle RNA (esRNA) in lymphoma. Crit Rev Oncol Hematol. (2019) 137:27–34. 10.1016/j.critrevonc.2019.01.013 31014513

[22] van Eijndhoven MA Zijlstra JM Groenewegen NJ Drees EE van Niele S Baglio SR. Plasma vesicle miRNAs for therapy response monitoring in Hodgkin lymphoma patients. JCI Insight. (2016) 1:e89631. 10.1172/jci.insight.89631 27882350 PMC5111516

[23] Feng Y Zhong M Zeng S Wang L Liu P Xiao X. Exosome-derived miRNAs as predictive biomarkers for diffuse large B-cell lymphoma chemotherapy resistance. Epigenomics. (2019) 11:35–51. 10.2217/epi-2018-0123 30211623

[24] Bortoluzzi S Bisognin A Biasiolo M Guglielmelli P Biamonte F Norfo R. Characterization and discovery of novel miRNAs and moRNAs in JAK2V617F-mutated SET2 cells. Blood. (2012) 119:e120–30. 10.1182/blood-2011-07-368001 22223824

[25] Agnelli L Bisognin A Todoerti K Manzoni M Taiana E Galletti S. Expanding the repertoire of miRNAs and miRNA-offset RNAs expressed in multiple myeloma by small RNA deep sequencing. Blood Cancer J. (2019) 9:21. 10.1038/s41408-019-0184-x 30783080 PMC6381125

[26] Bortoluzzi S Biasiolo M Bisognin A MicroRNA–offset RNAs (moRNAs): by-product spectators or functional players? Trends Mol Med. (2011) 17:473–4. 10.1016/j.molmed.2011.05.005 21700497

[27] Maute RL Schneider C Sumazin P Holmes A Califano A Basso K. tRNA-derived microRNA modulates proliferation and the DNA damage response and is down-regulated in B cell lymphoma. Proc Natl Acad Sci USA. (2013) 110:1404–9. 10.1073/pnas.1206761110 23297232 PMC3557069

[28] Asikainen S Heikkinen L Juhila J Holm F Weltner J Trokovic R. Selective microRNA-offset RNA expression in human embryonic stem cells. PLoS ONE. (2015) 10:e0116668. 10.1371/journal.pone.0116668 25822230 PMC4378994

[29] Nolte-'t Hoen ENM Buermans HPJ Waasdorp M Stoorvogel W Wauben MHM 't Hoen PAC. Deep sequencing of RNA from immune cell-derived vesicles uncovers the selective incorporation of small non-coding RNA biotypes with potential regulatory functions. Nucleic Acids Res. (2012) 40:9272–85. 10.1093/nar/gks658 22821563 PMC3467056

[30] Tosar JP Gámbaro F Sanguinetti J Bonilla B Witwer KW Cayota A. Assessment of small RNA sorting into different extracellular fractions revealed by high-throughput sequencing of breast cell lines. Nucleic Acids Res. (2015) 43:5601–16. 10.1093/nar/gkv432 25940616 PMC4477662

[31] Lunavat TR Cheng L Kim D-K Bhadury J Jang SC Lässer C. Small RNA deep sequencing discriminates subsets of extracellular vesicles released by melanoma cells–evidence of unique microRNA cargos. RNA Biol. (2015) 12:810–23. 10.1080/15476286.2015.1056975 26176991 PMC4615768

[32] Yuan T Huang X Woodcock M Du M Dittmar R Wang Y. Plasma extracellular RNA profiles in healthy and cancer patients. Sci Rep. (2016) 6:19413. 10.1038/srep19413 26786760 PMC4726401

[33] Gaffo E Zambonelli P Bisognin A Bortoluzzi S Davoli R. miRNome of Italian Large White pig subcutaneous fat tissue: new miRNAs, isomiRs and moRNAs. Anim Genet. (2014) 45:685–98. 10.1111/age.12192 25039998

[34] Langmead B Trapnell C Pop M Salzberg SL. Ultrafast and memory-efficient alignment of short DNA sequences to the human genome. Genome Biol. (2009) 10:R25. 10.1186/gb-2009-10-3-r25 19261174 PMC2690996

[35] Collado-Torres L Nellore A Frazee AC Wilks C Love MI Langmead B. Flexible expressed region analysis for RNA-seq with derfinder. Nucleic Acids Res. (2017) 45:e9. 10.1093/nar/gkw852 27694310 PMC5314792

[36] Love MI Huber W Anders S. Moderated estimation of fold change and dispersion for RNA-seq data with DESeq2. Genome Biol. (2014) 15:550. 10.1186/s13059-014-0550-8 25516281 PMC4302049

[37] Hendrick JP Wolin SL Rinke J Lerner MR Steitz JA. Ro small cytoplasmic ribonucleoproteins are a subclass of La ribonucleoproteins: further characterization of the Ro and La small ribonucleoproteins from uninfected mammalian cells. Mol Cell Biol. (1981) 1:1138–49. 10.1128/MCB.1.12.1138 6180298 PMC369740

[38] Lerner MR Boyle JA Hardin JA Steitz JA. Two novel classes of small ribonucleoproteins detected by antibodies associated with lupus erythematosus. Science. (1981) 211:400–2. 10.1126/science.6164096 6164096

[39] Mosig A Guofeng M Stadler BMR Stadler PF. Evolution of the vertebrate Y RNA cluster. Theory Biosci. (2007) 126:9–14. 10.1007/s12064-007-0003-y 18087752

[40] Teunissen SW Kruithof MJ Farris AD Harley JB Venrooij WJ Pruijn GJ. Conserved features of Y RNAs: a comparison of experimentally derived secondary structures. Nucleic Acids Res. (2000) 28:610–9. 10.1093/nar/28.2.610 10606662 PMC102524

[41] Maraia RJ Sasaki-Tozawa N Driscoll CT Green ED Darlington GJ. The human Y4 small cytoplasmic RNA gene is controlled by upstream elements and resides on chromosome 7 with all other hYscRNA genes. Nucleic Acids Res. (1994) 22:3045–52. 10.1093/nar/22.15.3045 7520568 PMC310274

[42] Sim S Wolin SL. Emerging roles for the Ro 60-kDa autoantigen in noncoding RNA metabolism. Wiley Interdiscip Rev RNA. (2011) 2:686–99. 10.1002/wrna.85 21823229 PMC3154076

[43] Christov CP Gardiner TJ Szüts D Krude T. Functional requirement of noncoding Y RNAs for human chromosomal DNA replication. Mol Cell Biol. (2006) 26:6993–7004. 10.1128/MCB.01060-06 16943439 PMC1592862

[44] Perreault J Perreault J-P Boire G. Ro-associated Y RNAs in metazoans: evolution and diversification. Mol Biol Evol. (2007) 24:1678–89. 10.1093/molbev/msm084 17470436

[45] Wei Z Batagov AO Schinelli S Wang J Wang Y El Fatimy R. Coding and noncoding landscape of extracellular RNA released by human glioma stem cells. Nat Commun. (2017) 8:1145. 10.1038/s41467-017-01196-x 29074968 PMC5658400

[46] Chakrabortty SK Prakash A Nechooshtan G Hearn S Gingeras TR. Extracellular vesicle-mediated transfer of processed and functional RNY5 RNA. RNA. (2015) 21:1966–79. 10.1261/rna.053629.115 26392588 PMC4604435

[47] Donovan J Rath S Kolet-Mandrikov D Korennykh A. Rapid RNase L–driven arrest of protein synthesis in the dsRNA response without degradation of translation machinery. RNA. (2017) 23:1660–71. 10.1261/rna.062000.117 28808124 PMC5648034

[48] Dhahbi JM Spindler SR Atamna H Boffelli D Mote P Martin DIK. 5′-YRNA fragments derived by processing of transcripts from specific YRNA genes and pseudogenes are abundant in human serum and plasma. Physiol Genomics. (2013) 45:990–8. 10.1152/physiolgenomics.00129.2013 24022222

[49] Dhahbi JM Spindler SR Atamna H Boffelli D Martin DIK. Deep sequencing of serum small RNAs identifies patterns of 5′ tRNA half and YRNA fragment expression associated with breast cancer. Biomark Cancer. (2014) 6:BIC.S20764. 10.4137/BIC.S20764 25520563 PMC4260766

[50] Yeri A Courtright A Reiman R Carlson E Beecroft T Janss A. Total extracellular small RNA profiles from plasma, saliva, and urine of healthy subjects. Sci Rep. (2017) 7:44061. 10.1038/srep44061 28303895 PMC5356006

[51] Solé C Tramonti D Schramm M Goicoechea I Armesto M Hernandez LI. The circulating transcriptome as a source of biomarkers for melanoma. Cancers. (2019) 11:E70 10.3390/cancers11010070 30634628 PMC6356785

[52] Li C Qin F Hu F Xu H Sun G Han G. Characterization and selective incorporation of small non-coding RNAs in non-small cell lung cancer extracellular vesicles. Cell Biosci. (2018) 8:2. 10.1186/s13578-018-0202-x 29344346 PMC5763536

[53] Haderk F Schulz R Iskar M Cid LL Worst T Willmund KV. Tumor-derived exosomes modulate PD-L1 expression in monocytes. Sci Immunol. (2017) 2:eaah5509. 10.1126/sciimmunol.aah5509 28754746

[54] Verhagen APM Pruijn GJM. Are the Ro RNP-associated Y RNAs concealing microRNAs? Y RNA-derived miRNAs may be involved in autoimmunity. Bioessays. (2011) 33:674–82. 10.1002/bies.201100048 21735459

[55] Langenberger D Çakir MV Hoffmann S Stadler PF. Dicer-processed small RNAs: rules and exceptions. J Exp Zool B Mol Dev Evol. (2013) 320:35–46. 10.1002/jez.b.22481 23165937

[56] Thomson DW Pillman KA Anderson ML. Assessing the gene regulatory properties of Argonaute-bound small RNAs of diverse genomic origin. Nucleic Acids Res. (2014) 43:470–81. 10.1093/nar/gku1242 25452337 PMC4288155

[57] Köhn M Ihling C Sinz A Krohn K Hüttelmaier S The Y3^**^ ncRNA promotes the 3′ end processing of histone mRNAs. Genes Dev. (2015) 29:1998–2003. 10.1101/gad.266486.115 26443846 PMC4604341

[58] Driedonks TAP van der Grein SG Ariyurek Y Buermans HPJ Jekel H Chow FWN. Immune stimuli shape the small non-coding transcriptome of extracellular vesicles released by dendritic cells. Cell Mol Life Sci. (2018) 75:3857–75. 10.1007/s00018-018-2842-8 29808415 PMC6154026

[59] Driedonks TAP Nolte-'t Hoen ENM. Circulating Y-RNAs in extracellular vesicles and ribonucleoprotein complexes; implications for the immune system. Front Immunol. (2018) 9:3164. 10.3389/fimmu.2018.03164 30697216 PMC6340977

[60] Godoy PM Bhakta NR Barczak AJ Cakmak H Fisher S MacKenzie TC. Large differences in small RNA composition between human biofluids. Cell Rep. (2018) 25:1346–58. 10.1016/j.celrep.2018.10.014 30380423 PMC6261476

[61] Shurtleff MJ Yao J Qin Y Nottingham RM Temoche-Diaz MM Schekman R. Broad role for YBX1 in defining the small noncoding RNA composition of exosomes. Proc Natl Acad Sci USA. (2017) 114:E8987–95. 10.1073/pnas.1712108114 29073095 PMC5663387

[62] Qin Y Yao J Wu DC Nottingham RM Mohr S Hunicke-Smith S. High-throughput sequencing of human plasma RNA by using thermostable group II intron reverse transcriptases. RNA. (2016) 22:111–28. 10.1261/rna.054809.115 26554030 PMC4691826

[63] Tolkach Y Stahl AF Niehoff E-M Zhao C Kristiansen G Müller SC. YRNA expression predicts survival in bladder cancer patients. BMC Cancer. (2017) 17:749. 10.1186/s12885-017-3746-y 29126388 PMC5681827

[64] Lamant L McCarthy K d'Amore E Klapper W Nakagawa A Fraga M. Prognostic impact of morphologic and phenotypic features of childhood ALK-positive anaplastic large-cell lymphoma: results of the ALCL99 study. J ClinOncol. (2011) 29:4669–76. 10.1200/JCO.2011.36.5411 22084369

